# A synthetic coolant (WS-23) in disposable electronic cigarettes impairs cytoskeletal function in EpiAirway microtissues exposed at the air liquid interface

**DOI:** 10.1038/s41598-023-43948-4

**Published:** 2023-10-07

**Authors:** Man Wong, Teresa Martinez, Mona Tran, Cori Zuvia, Alisa Gadkari, Esther E. Omaiye, Wentai Luo, Kevin J. McWhirter, Jihui Sha, Ahmad Kassem, James Wohlschlegel, Prue Talbot

**Affiliations:** 1 Department of Molecular, Cell and Systems Biology, University of California, Riverside, CA 92521 USA; 2 Department of Civil and Environmental Engineering, Portland State University, Portland, OR 97207 USA; 3 Department of Biological Chemistry, David Geffen School of Medicine at University of California, Los Angeles, CA 90095 USA

**Keywords:** Respiratory tract diseases, Cell adhesion, Cell growth, Cell migration, Cell signalling, Cytoskeleton

## Abstract

The design of popular disposable electronic cigarettes (ECs) was analyzed, and the concentrations of WS-23, a synthetic coolant, in EC fluids were determined for 22 devices from 4 different brands. All products contained WS-23 in concentrations that ranged from 1.0 to 40.1 mg/mL (mean = 21.4 ± 9.2 mg/mL). To determine the effects of WS-23 on human bronchial epithelium in isolation of other chemicals, we exposed EpiAirway 3-D microtissues to WS-23 at the air liquid interface (ALI) using a cloud chamber that generated aerosols without heating. Proteomics analysis of exposed tissues revealed that the cytoskeleton was a major target of WS-23. BEAS-2B cells were exposed to WS-23 in submerged culture to validate the main results from proteomics. F-actin, which was visualized with phalloidin, decreased concentration dependently in WS-23 treated BEAS-2B cells, and cells became immotile in concentrations above 1.5 mg/mL. Gap closure, which depends on both cell proliferation and migration, was inhibited by 0.45 mg/mL of WS-23. These data show that WS-23 is being added to popular EC fluids at concentrations that can impair processes dependent on the actin cytoskeleton and disturb homeostasis of the bronchial epithelium. The unregulated use of WS-23 in EC products may harm human health.

## Introduction

Synthetic coolants (WS-3 and WS-23), which were developed by Wilkerson-Sword in the 1950s for use in consumer products such as shaving cream^1^, were recently introduced into fourth generation electronic cigarette (EC) liquids (e-liquids)^2–5^. Originally reported in low concentrations in JUUL pods^3,4^, synthetic coolants were subsequently found in much higher concentrations in Puff products^4–6^. WS-23 concentrations in Puff e-liquids were as high as 40 mg/mL^4^ and exceeded concentrations in other consumer products, such as candy, beverages, and gum^7^. While WS-23 is often used in ECs with “icy” or “cool” flavor names, such as Puff “Cool Mint” or “Lychee Ice”, it is also used in flavors that do not imply icy or coolness, such as Puff “Tobacco” and “Mixed Berries^4^”. JUUL ECs dominated the early fourth generation EC market^8^, but were superseded by Puff and other disposable brands, which were not initially affected by the Food and Drug Administration’s (FDA) restrictive enforcement policy on flavors in cartridge style ECs^9^. Because Puff ECs were disposable, not cartridge style, they were able to offer JUUL users a range of attractive flavors^10^.

ECs have evolved rapidly in design, fluid chemistry, and popularity^4,11^, and many new brands now compete with Puff. As more disposable EC devices were introduced into the market, Puff products became less popular, and many local retailers stopped carrying them. In southern California, the most popular disposable fourth generation brands are currently Flum and ELFBAR, which are both available in a range of popular flavors that have not been analyzed previously for e-liquid chemistry or toxicity. The purpose of this study was to quantify the synthetic coolants (WS-3 and WS-23) in Flum Float, ELFBAR, Puff XL, Puff Plus, and Puff Bar Plus e-liquids and to evaluate toxicity of WS-23 using 3D cultures of human bronchial epithelium (EpiAirway) that were exposed to WS-23 at the air–liquid interface (ALI) in a cloud chamber. The dominant effects of WS-23 on exposed EpiAirway were determined using proteomics analysis followed by relevant in vitro assays.

## Results

### Characterization of popular 4th generation disposable ECs that deliver high puff numbers

Websites and online forums were searched to identify the most popular disposable ECs on the market in 2022; these are shown in Supplementary Table 1. Popular brands have evolved to deliver 3000 to 10,000 puffs per EC. For ECs delivering > 3,000 puffs, the volume of e-liquid/device ranged from 6.5 to 20 mL. Power was variable and ranged from 400 to 1,500 mAh. All products in Supplementary Table 1 were sold as disposable, and 23 of 36 were rechargeable. Some products were a part of the same product line with puff number and e-liquid volume being the only difference (e.g., ELFBAR BC5000 and ELFBAR BC3500).

From these devices, we selected Flum Float, ELFBAR BC5000, and Puff Xtra Limited for further analysis. In addition, we also analyzed Flum Gio, Puff Plus, and Puff Bar Plus (predecessor of Puff Plus). Although not identified as “popular” on websites and forums, Puff Bar was one of the first disposable EC brands to dominate the EC market. While Puff Bars popularity has declined recently, it was included for comparison to the newer disposable products. Flum Gio and Flum Pebble were included as upgraded versions of the Flum Float.

The Flum Float and ELFBAR BC5000 were the most popular ECs in local shops and online. Puff XTRA Limited (a different brand than Puff Bar) was popular in our local vape shop. The Flum Gio is a slightly updated version of the Flum Float, with the same puff number and e-liquid volume. The Flum Pebble was released near the end of 2022 with upgraded features that include a higher puff number, larger e-liquid volume, and a rechargeable battery. ELFBAR had the most variety in its product line, with over 20 different devices.

**Figure 1:**
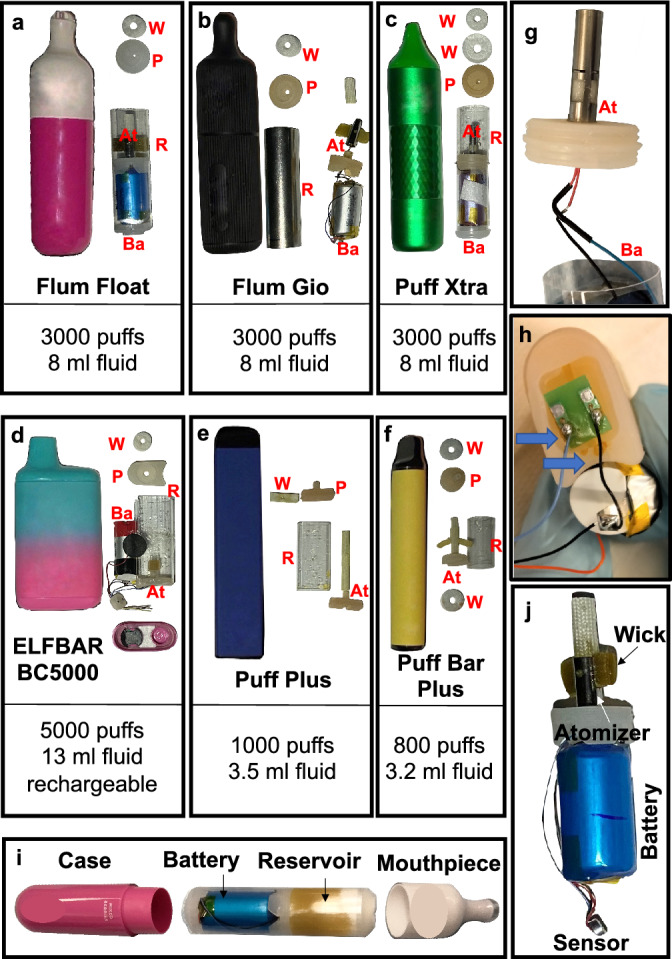
Dissections of six disposable ECs. (**a**) Flum Float, (**b**) Flum Gio, (**c**) Puff Xtra Limited, (**d**) ELFBAR BC5000, (**e**) Puff Plus, and (**f**) Puff Bar Plus. (**a**–**f**) show the EC before dissection and the battery and tank/atomizer after removal from the case. (**g**) A zoomed image of a Flum Float atomizer. (**h**) ELFBAR BC5000 showing signs of leakage (blue arrows) after 65 puffs. (**i**) Flum Float disassembled before dissection and (**j**) Battery and atomizer component of Flum Float after dissection. At—Atomizer, Ba—Battery, R—Reservoir, W—Washer, P—Plug.

Flum Float, Flum Gio, Puff Xtra Limited, ELFBAR BC 5000, Puff Plus, and Puff Bar Plus were dissected (Fig. 1). While the devices were different externally, the internal components were similar across products and were distinctly different from brands examined previously^4,11,12^. All devices had a mouthpiece and a case enclosing a reservoir (R), which contained a sponge, an atomizer (At), and a battery (B) (Fig. 1a,b,c,d,e,f,g,i). In both the Puff Plus and Puff Bar Plus, the batteries were attached to the case and could not be removed without damaging the atomizer. As a result, some components are missing in the figures showing Puff devices (Fig. 1e,f).

The atomizers were made of metal, except in ELFBAR BC5000 “Tropical Rainbow Blast”, which had a plastic atomizer (see Supplementary Figure S1 online). In the Flum Float “Cool Mint” and “Mixed Berries”, the tank and battery components were attached forming a single unit, while in “Blue Raspberry Ice” and “Aloe Mango Melon Ice”, the tank and battery were separate (see supplementary Figure S2 online). The Flum Float and Flum Gio designs were similar, however some materials used in the devices were different. The Flum Float had a plastic exterior, while the exterior of the Flum Gio was silicone, which may be easier to grip and may have a more comfortable mouthpiece. The atomizer and battery were encased in plastic in the Flum Float and in metal in the Flum Gio (Fig. 1a,b). The Flum Gio was lighter in weight than the Flum Float, and the available flavors differed between the two products.

The design features of the disposable products in the current study differed from other 4th generation pod-style ECs in two ways: (1) each had a non-removable reservoir that contained a sponge loaded with e-fluid instead of a pod or tank that contained e-fluid, and (2) the reservoir contained sufficient e-liquid to enable very high puff numbers with a single device.

### Disposable ECs contained high concentrations of the synthetic coolant WS-23

**Figure 2:**
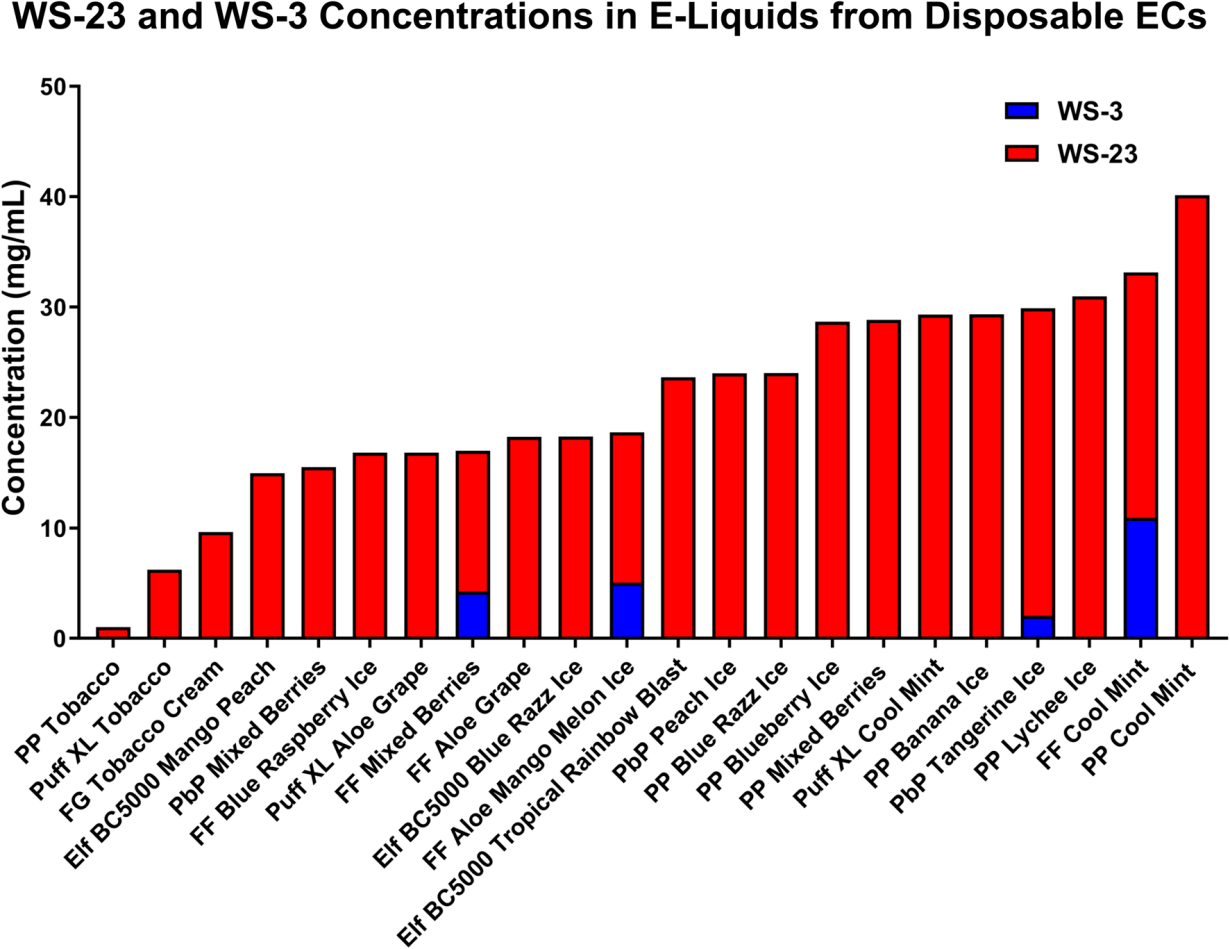
WS-3 and WS-23 concentrations in 22 popular EC products. Actual concentrations of WS-23 and WS-3 can be read directly off the graph. WS-23 was detected in all devices and ranged from 1.0 mg/mL (Puff Plus “Tobacco”) to 40.1 mg/mL (Puff Plus “Cool Mint”). The average WS-23 concentration was 21.4 mg/ml ± 9.21 mg/ml. WS-3 was found in four samples at concentrations ranging from 2.05 mg/ml (Puff Bar Plus “Tangerine Ice”) to 10.93 mg/ml (Flum Float “Cool Mint”). PP—Puff Plus, FF—Flum Float, Elf—ELFBAR, PbP—Puff Bar Plus.

WS-23 and WS-3 were quantified in each product. WS-23 was detected in every product and ranged from 1.0 mg/mL (Puff Plus “Tobacco”) to 40.1 mg/mL (Puff Plus “Cool Mint”) (Fig. 2, Supplemental Table 2) The average WS-23 concentration across all products was 21.4 mg/mL ± 9.2 mg/mL. WS-3 was found in only four samples at concentrations ranging from 2 mg/ml (Puff Bar Plus “Tangerine Ice”) to 10.9 mg/mL (Flum Float “Cool Mint”). WS-3 was detected in four other samples below the limit of quantification (Supplemental Table 3). Because WS-23 was present in all samples, often at high concentrations, it was used for subsequent ALI exposures of EpiAirway microtissues and submerged exposures in the BEAS-2B experiments.

### Proteomics of EpiAirway exposed to WS-23 reveal the cytoskeleton as a major target

**Figure 3:**
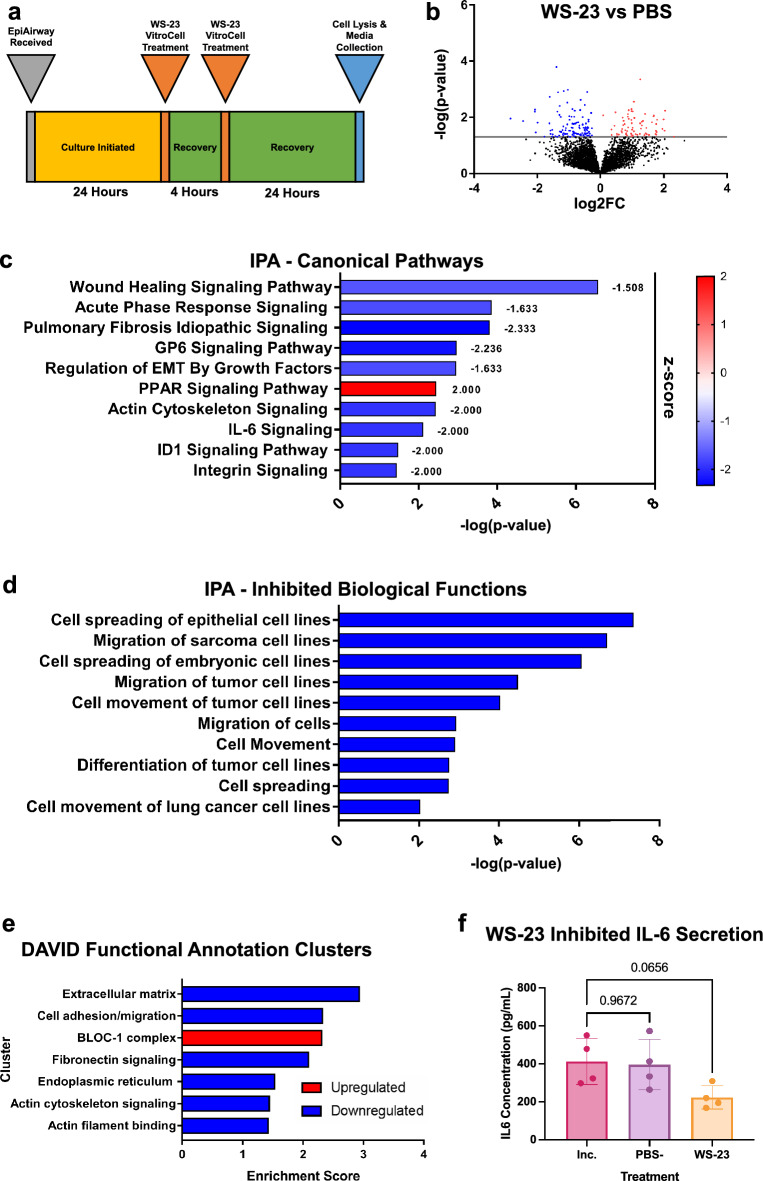
Proteomics analysis of EpiAirway microtissues treated with WS-23 in a cloud chamber. (**a**) Schematic showing experimental design. (**b**) Volcano plot showing the proteins that were significantly different in the WS-23 versus the PBS control comparison. Red and blue are up and down regulated, respectively. (**c**) IPA Canonical Pathway Analysis with z-score labeled next to each bar. (**d**) IPA Biological Functions Analysis showing processes that were significantly inhibited by WS-23. (**e**) DAVID clusters from significantly upregulated (red) and downregulated (blue) proteins. (**f**) Concentrations of IL-6 in EpiAirway culture medium 24 h after exposure to WS-23. Data are the means ± standard deviations of four independent experiments. Means were compared using an ANOVA followed by Dunnett’s posthoc test with PBS being the comparator group. *P* values are shown above bars.

To obtain a global perspective on the targets of WS-23, the proteome was characterized in EpiAirway microtissues following ALI exposure to WS-23 in a cloud chamber. EpiAirway were exposed to 2 nebulized puffs of WS-23 with a 4 h interval between puffs, then allowed to recover 24 h before lysis and proteomics analysis (Fig. 3a). Each puff delivered roughly 7.5 ± 0.47 µg of WS-23 to each well. Proteins that were significantly decreased or increased in the WS-23 treated group vs the PBS control group are shown in the volcano plot (Fig. 3b). A total of 182 proteins were significantly affected with 70 upregulated and 112 downregulated when compared to the PBS control.

IPA analysis identified 10 canonical pathways (Z-scores > 1.5) that were targets of WS-23, of which 9 were downregulated and one (PPAR Signaling) was upregulated. Downregulated pathways generally involved the cytoskeleton and cytokine secretion/response and included: Wound Healing Signaling, Acute Phase Response Signaling (rapid reprogramming of gene expression and metabolism in response to inflammatory cytokine signaling), Pulmonary Fibrosis Idiopathic Signaling Pathway, Regulation of EMT by Growth Factors Pathway, Actin Cytoskeleton Signaling, and IL-6 Signaling (Fig. 3c). IPA Biological Functions Analysis included inhibition in cell spreading, migration, and movement (Fig. 3d). Downregulation of IL-6 signaling was confirmed using ELISAs to quantify the levels of IL-6 in the EpiAirway medium, which was collected 24 h after exposure to WS-23 (Fig. 3f).

DAVID analysis of upregulated proteins yielded one significant cluster, the BLOC-1 complex, which is involved in endosomal trafficking and maturation in melanocytic cells by creating molecular linkages between actin and microtubule cytoskeletons^13^. DAVID analysis of downregulated proteins yielded six significant clusters, which included extracellular matrix, cell adhesion/migration, fibronectin, endoplasmic reticulum, actin cytoskeletal signaling, and actin filament binding, implicating the cytoskeleton as a major target (Fig. 3e). There was good agreement between the IPA and DAVID analyses, with both identifying actin cytoskeleton signaling as a target of WS-23, including downstream effects such as cell spreading, cell adhesion, cell migration, and cell movement.

**Figure 4:**
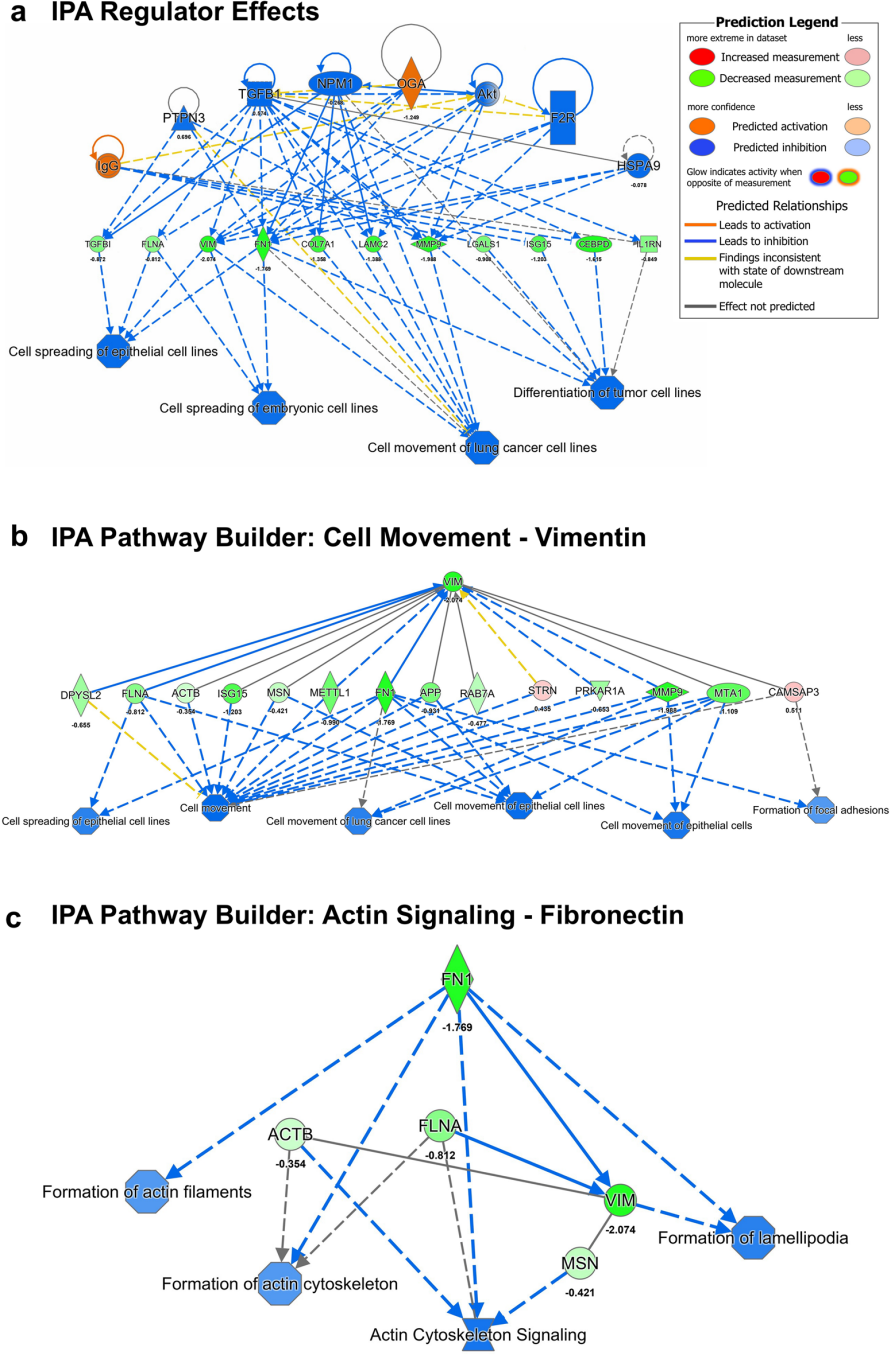
IPA analysis of predicted pathways affected by WS-23. Proteins measured in our samples are shown in red (upregulated) or green (downregulated); based on these changes, IPA predicted if other proteins would be activated (orange) or inhibited (blue). (**a**) Regulator Effects Analysis shows upstream regulators, such as TGFB1, OGA, and Akt; targets of the regulators that were affected in our data (middle row) and the predicted phenotypical or functional outcomes, such as cell movement and cell spreading. (**b** and **c**) Pathway Builder Analysis for Vimentin and Fibronectin, respectively. Pathway Builder generated pathways from specific proteins from our dataset. In both analyses, proteins that were affected were predicted to decrease processes dependent of the cytoskeleton.

The Regulatory Effects Analysis in IPA was used to predict upstream regulators and downstream processes affected by changes in the proteins in our data (Fig. 4a). Processes predicted to be downregulated included Cell Spreading in Epithelial Lines, Cell Spreading in Embryonic Stem Cells, Cell Movement, and Differentiation of Tumor Cells. The IPA Pathway Builder Tool was used to build pathways that linked specific proteins in our data set to a downstream process or function. This tool allows us to visualize interactions that a specific protein may have with other proteins in a pathway and how they are connected to a particular process. Pathways were seeded off vimentin, which had decreased (green) expression and was a major target for many of the proteins that changed in our dataset. Many of the motility and cell spreading processes affected by WS-23 treatments were downstream of vimentin (Fig. 4b). Fibronectin was also decreased by WS-23 treatment and was upstream of actin cytoskeleton signaling (Fig. 4c). The dominant effects of WS-23 observed in the proteomics data involved decreased activity in processes involving the cytoskeleton, cell motility, and cell spreading.

### WS-23 causes actin to depolymerize and inhibits cell motility and attachment in BEAS-2B cells

**Figure 5:**
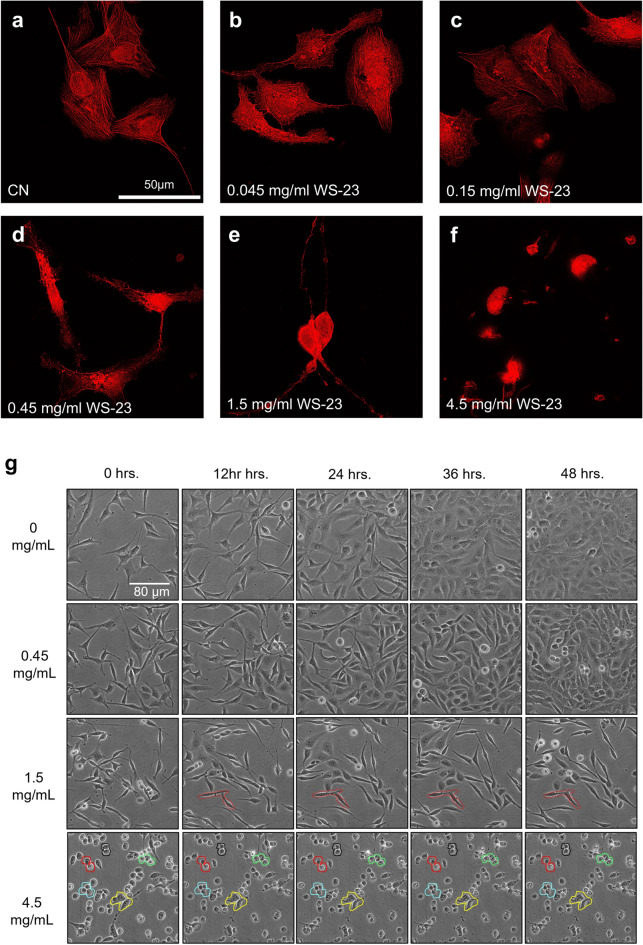
BEAS-2B cells labeled with phalloidin following WS-23 treatment in submerged culture. (**a**–**f**) Fluorescent images of cells treated with varying concentrations of WS-23 then labeled with phalloidin. (**g**) Live cell images captured in a BioStation CT during treatment with WS-23. Cells circled in colors did not move during the treatment period. Concentrations are those present in the e-liquid before aerosolization.

The effects of WS-23 exposure were examined further using in vitro assays specific to the processes predicted with DAVID and IPA. Assays were performed using BEAS-2B cells cultured in submerged monolayers, which we showed previously produce results similar to those obtained with ALI exposure in a cloud chamber^14^. BEAS-2B cells in monolayer culture were treated with various concentrations of WS-23 for 8 h and then labeled with phalloidin, a fungal peptide that binds specifically to F-actin^15,16^. Untreated cells were well spread and had numerous F-actin filaments (stress fibers) in their cytoplasm (Fig. 5a). At 0.045 mg/mL of WS-23, cells had fewer F-actin filaments and the fluorescent signal was more diffuse rather than concentrated in stress fibers (Fig. 5b). As the concentration of WS-23 increased, cells were less well spread and there was less stabilized F-actin and more diffuse signal, indicating the actin was depolymerizing (Fig. 5c). At 0.45 mg/mL, the cells were smaller and phalloidin signal was more intense compared to the control (Fig. 5d). At 1.5 mg/mL, cells began to round and were no longer spread properly (Fig. 5e). At the highest WS-23 concentration (4.5 mg/mL), cells were rounded, and actin was concentrated in the cell center (Fig. 5f). These data demonstrated impairment of cell spreading accompanied by rounding due to a concentration-dependent collapse of the actin cytoskeleton.

Live cell imaging in a BioStation CT was used to evaluate the motility of BEAS-2B cells exposed to various concentrations of WS-23 (Fig. 5g). At 1.5 mg/mL of WS-23, cells became less motile throughout the course of the treatment (colored outlines), and at 4.5 mg/mL of WS-23, cells rounded, did not spread normally, and did not move during 48 h of culture. While 0.45 mg/ml did not seem to significantly impact motility, control cells were flatter (white halos surrounding control cells were less pronounced and control cells were less phase dense than those treated with 0.45 mg/ml of WS-23), indicating the treated cells were not spreading or attaching as efficiently as the control cells (Fig. 5g).

### WS-23 inhibited the ability of BEAS-2B cells to perform the gap closure

**Figure 6:**
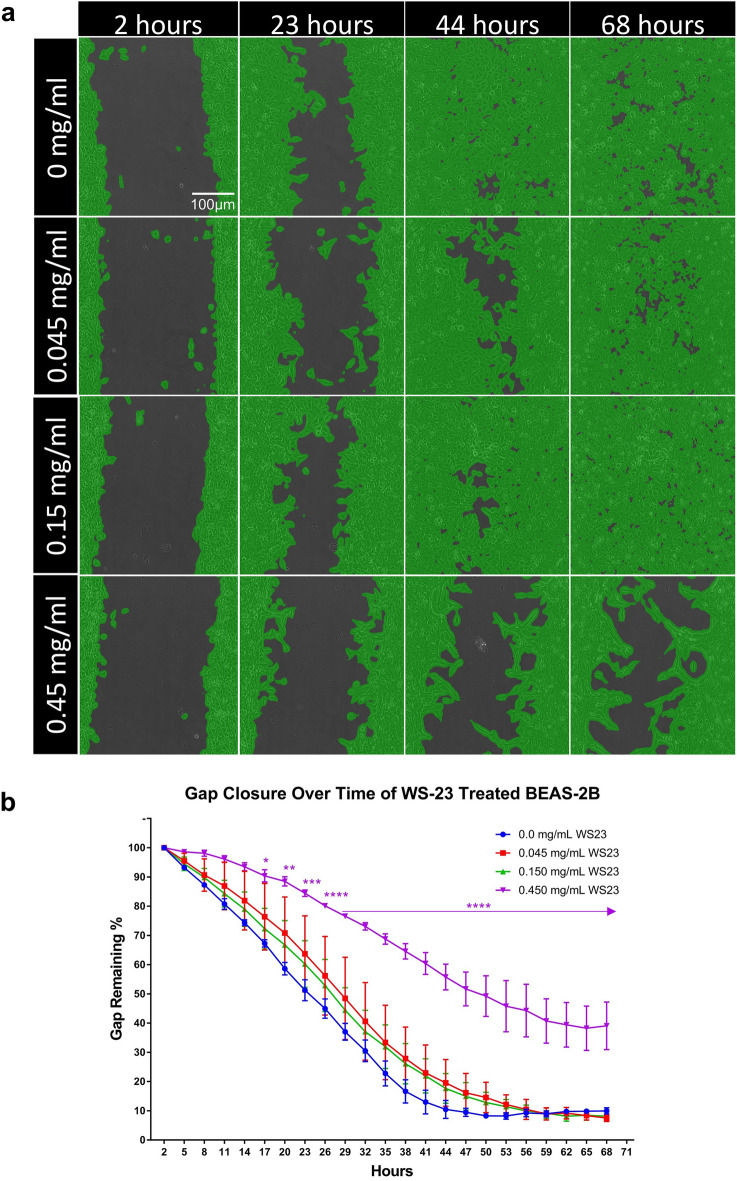
Effect of WS-23 on gap closure of BEAS-2B cells. (**a**) Time lapse images of gap closure during WS-23 treatment over 68 h. (**b**) Graph showing area of gap through 68 h of exposure. Concentrations are those present in the e-liquid before aerosolization. Each point in B is the mean ± standard error of the mean of three experiments. Data were analyzed using a 2-way ANOVA followed by Dunnett’s posthoc test. *p < 0.05, **p < 0.01, ***p < 0.001, ****p < 0.0001.

A gap closure assay was performed to evaluate the effect of WS-23 on motility in a monolayer of BEAS-2B cells (Fig. 6a,b). Over the course of 68 h, BEAS-2B cells treated with 0.45 mg/mL of WS-23 were unable to completely close the gap, in contrast to controls, which closed the gap by 44 h. A significant decrease in the rate of gap closure was seen in the 0.15 mg/mL group beginning at 17 h (Fig. 6b). All other treatments produced results similar to the untreated control.

## Discussion

The disposable ECs in our study contained design features that distinguish them from prior generations^11^. These features included: (1) increases in the number of puffs delivered/EC, as reported by the manufacturers; (2) addition of very high concentrations of WS-23 to many disposable products; WS-23 concentrations were often higher than the flavor chemicals reported previously, which were considered high for consumer products^5,17^; (3) a closed tank that is not intended to be changed, accessed, or refilled; (4) a “sponge” like material in the atomizer that absorbs e-fluid and holds it near the filament; the sponge may reduce leaking of fluid; and (5) the ability to recharge some of the newer disposable products. The internal design was remarkably similar in two different brands (Flum and ELFBAR), which are marketed by different companies. There are sufficient differences between the disposable ECs in this study and reusable pod style 4th generation products, such as JUUL^4^, that the disposable ECs may deserve their own generational category (5th generation or rechargeable disposable).

Disposable 4th generation ECs can deliver very high puff numbers, and in our samples, most were capable of > 3,000 puffs/device, in contrast to JUUL, which delivers ~ 200 puffs/pod. Some disposable ECs can be recharged, probably to refresh the battery so it can deliver high puff numbers. Many of the components in the disposable atomizers were made with metals and/or plastics that could leach chemicals into the e-liquid with use^12,18,19^. The health effects of vaping thousands of puffs from a single EC are unknown, but should be investigated since residues, corrosion, and leaching of chemicals and metals from atomizer components could occur during repeated cycles of heating and cooling. As previously shown, metal leachates in e-liquids tended to be higher in first generation ECs that were heavily used when compared to those that were gently used^18^. As the demand for higher puff numbers and rechargeable devices increases, the puff numbers for disposable ECs may go even higher. The Flum Float and Gio were designed to deliver around 3,000 puffs/EC and are not rechargeable, in contrast to their successor Flum Pebble (launched late 2022), which can deliver 6,000 puffs and is rechargeable. In early 2023, ELFBAR also released two upgraded products that surpassed the puff number of ELFBAR BC5000. Due to a trademark lawsuit against VPR Brands^20^, ELFBAR rebranded in the US as EBDESIGN in March of 2023. EBDESIGN Pi7000 and EBDESIGN TE6000 are rechargeable and reach 7000 and 6000 puffs, respectively. As was recently shown, the number of symptoms reported by JUUL users increased with increasing puff number^21^, suggesting these newer devices with higher puff numbers may be more likely to increase adverse health effects than earlier generation products that deliver low puff numbers.

The addition of synthetic coolants to e-fluids is another industry trend. Early 4th generation products, such as JUUL Menthol, had low concentrations of synthetic coolants (0.1 mg/mL WS-23)^6^. Disposable EC products have much higher concentrations of coolants than JUUL pods. All products in the current study had at least 1 mg/mL of WS-23, and most had concentrations above 10 mg/mL. As reported previously^4^, synthetic coolants were present in e-liquids in disposable products that were not characterized as “Cool” or “Ice” (e.g., Flum Gio “Tobacco Cream”, ELFBAR BC5000 “Mango Peach”, and Flum Float “Aloe Grape”). The FEMA GRAS status of WS-23 pertains to ingestion, not inhalation exposure^22^. Most studies that have examined WS-23 toxicity have used rodent models with oral exposure. One rat study that evaluated inhalation of WS-23 found few acute or subacute effects^23^. However, their exposures were below those that would be received by a human vaping WS-23 at the concentrations present in the disposable ECs in our study. In submerged cultures, pure WS-23 produced cytotoxicity in the MTT assay at concentrations well below those in disposable ECs^6^. Margin of exposure (MOE) analysis estimates risk by predicting the likelihood that a chemical would produce adverse health effects. In various scenarios, the MOE for WS-23 was below the threshold of 100 indicating that it is likely to adversely affect health^4^. Disposable ECs are attractive to users, simple, cheap, and do not require maintenance, which will continue to contribute to their appeal. As disposable ECs evolve towards more powerful devices with higher puff numbers, their health and environmental impacts will need to be carefully evaluated.

As ECs rapidly evolve, regulatory agencies, such as the FDA, are constantly adapting to keep up with manufacturers’ innovations. When JUUL was popular with youth, the FDA issued an enforcement policy that significantly reduced the sales of flavored 4th generation pod/cartridge ECs^9^. However, disposable ECs, which were not covered by the enforcement policy, such as Puff Bar, rapidly gained popularity among young vapers^8,10^. In July of 2020, the FDA issued warning letters to 10 companies, including Puff Bar, to remove their flavored disposable EC products from the market^24^, prompting Puff to cease online sales and distribution in the US between July 2020 to January 2021 (Puffbar.com). However, between February 2021 to December 2022, Puff sold ECs containing synthetic nicotine, which at the time was not regulated by the FDA.

When the FDA was given jurisdiction over synthetic nicotine-containing products, Puff again removed ECs from their website, which likely contributed to the rise in popularity of ELFBAR and Flum. In December 2022, Puff reactivated their online sales offering only zero nicotine ECs. Zero nicotine appears to be an industry trend that Puff and other companies (e.g., Flum and ELFBAR/EBDESIGN) are incorporating into their products.

As of December 2022, ELFBAR was the third top selling EC brand in America and top selling disposable EC brand^25^. In June 2023, the FDA sent warning letters to retailers regarding the illegal sales of ELFBAR and Esco Bar products^26^. As the FDA cracks down on ELFBAR/EBDESIGN products, it will be important to pay attention to how industry trends and popularity shift. Flum is another popular disposable EC brand that may be primed to overtake ELFBAR as the top selling disposable EC. Given the rapidity with which ECs evolve, there may be additional chemicals or coolants that become industry standards, further increases in puff numbers, or a completely new generation that will emerge from current disposable ECs. Researchers and regulatory agencies may struggle to keep up with changing trends and identifying chemicals or EC constituents that may require regulation. The current study demonstrates that regulatory issues exist, such as the high concentrations of coolants and flavor chemicals in EC fluids, although the regulatory authority of the FDA over ECs that lack nicotine may hamper regulation.

The generation of aerosol in a VitroCell cloud chamber allowed pure WS-23 to be examined independently of reaction products that form during heating in an EC. Our proteomics analysis of EpiAirway exposed to WS-23 at the ALI identified the cytoskeleton and cytoskeletal dependent processes as the major targets of pure WS-23. Both DAVID and IPA predicted a downregulation or inhibition in “actin cytoskeleton signaling”, along with other related pathways such as “cell spreading” and “cell movement”. With conservative exposures, there were 182 proteins that significantly changed in the proteomics analysis. Many of the proteins were related to actin signaling and could indicate that over time, users that vape disposable ECs may develop issues with the cytoskeleton in their bronchial epithelial cells. Cigarette smoke is also reported to elicit negative effects on the actin cytoskeleton and alter epithelial cell–cell adhesion, potentially contributing to COPD^27^. Disruptions to actin cytoskeletal homeostasis are likely to affect many other processes critical for bronchial epithelium function, such as ciliary motility, cell adhesion, and mucus and cytokine secretion. The agreement between DAVID and IPA further supports the conclusion that WS-23 is disrupting cytoskeleton homeostasis. Many of the clusters in DAVID and the Canonical Pathways and Biological Functions in IPA show that actin cytoskeleton signaling is inhibited by pure WS-23, in addition to downstream processes, such as wound healing, pulmonary fibrosis, cell adhesion, cell movement, cell spreading, and many others.

Proteomics data were supported by in vitro assays that evaluated actin polymerization, cell motility, cell spreading, IL-6 signaling, and gap closure. These data suggest that vapers who use products with high concentrations of WS-23 may have disruptions in actin signaling in their bronchial epithelial cells, and with prolonged use may experience destabilization of actin filaments and changes in cell or tissue morphology. Live cell imaging previously showed that BEAS-2B cell growth is inhibited in vitro by concentrations of WS-23 as low as 0.045 mg/mL^6^. We performed similar experiments but instead evaluated motility, spreading, and gap closure. The efficiency of gap closure was significantly inhibited in 0.45 mg/mL WS-23 beginning at the 17 h mark, which likewise supports the proteomics data. The decrease in IL-6 signaling that was predicted in the canonical pathway analysis done in IPA was supported by the in vitro experiments in which a decrease close to statistical significance was found (*p* = 0.065) in EpiAirway tissues exposed to WS-23. These data support the conclusion that EC users who vape WS-23 may experience effects on processes involving the cytoskeleton, cytokine secretion, and recovery from injury.

Based on data on chemical intake and retention in 11 JUUL users^21^, we estimated the exposure to WS-23 that EC users would receive based on WS-23 concentrations in our study. Using the average WS-23 concentration in our study (21.42 mg/ml) and assuming a 100% transfer efficiency, about 33.64 (± 14.54) µg of WS-23 would be absorbed by users/puff. Our two-puff exposure protocol in the cloud chamber delivered 15 µg of WS-23 to each EpiAirway insert, indicating our results are likely to be relevant to an average EC user vaping disposables ECs.

Our data define the effects of pure WS-23 aerosol, generated in a cloud chamber without heating, on EpiAirway tissues exposed at the ALI. Aerosols generated from e-liquid in an EC are generally complex mixtures that include solvents, flavor chemicals, nicotine, acids, metals, and reaction products^12,17,28–34^. We are currently determining if the effects of WS-23 are modulated when exposures are done using EC generated aerosols containing WS-23.

## Conclusions

The current sample of disposable ECs had design features that differed from earlier 4th generation pod-style products, such as JUUL. Within our sample set, designs of FLUM and ELFBAR products were similar and may represent a 5th generation of products. WS-23 was present in all products, usually at high concentrations (> 10 mg/mL), even when they were not designated ‘cool” or “icy”. Pure WS-23 exposure of 3D EpiAirway tissues at the ALI affected cytoskeletal-dependent processes, including cell motility, cell spreading, actin cytoskeleton signaling, lamellipodia formation, and wound healing. These processes were confirmed using in vitro cultures of BEAS-2B cells. WS-23 produces a cooling effect without imparting a flavor, which may make it attractive to EC manufacturers. The very high concentrations of WS-23 that in current products coupled with their demonstrated effects on cell homeostasis indicate that the use of synthetic coolants in ECs needs regulatory oversight.

## Methods

### Identification of popular disposable EC products

A Google search was done in the fall of 2022 using various key words (e.g., most popular disposable vapes in 2022) to identify the most popular disposable ECs on the market. Websites hosted by EC manufacturers were not included, as these were biased toward their products. Five websites were included, and each site had 8–12 ECs that fit the inclusion criteria. These sites were: https://provape.com/blog/post/; https://vaping360.com/best-beginner-e-cigs-; https://www.ejuices.com/blogs/ejuices/best-disposable-vapes-of-2022; https://www.eightvape.com/blogs/news/top-10-best-disposables-vapes; and https://versedvaper.com/best-disposable-e-cigs/. While Puff devices were not mentioned in the websites, we included them in our study since they were the first disposable EC to dominate the 4th generation EC market and many previous studies on disposable ECs looked at Puff products. In addition, we spoke with EC users and vape shop owners to identify the most popular brands in southern California. Based on the search and information provided by shop owners, four brands with different flavors were purchased for testing.

### Product acquisition

Disposable ECs were purchased at Select Smoke Shop Tarzana (Tarzana, CA) and Glassroots Smoke Shop (Riverside, CA). Both stores were authorized to sell Puff products (https://puffbar.com/pages/store-locator [webpage now inactive]) and other EC brands. To avoid the use of counterfeit ECs^35^, all products were verified to be authentic with either QR codes or serial numbers, except PuffXtra Limited (the device does not come with a QR code or serial number). Products were selected based on local popularity and sales volume in the stores where they were purchased. Five different EC devices from three manufacturers were evaluated: Puff Bar Plus (Vape Check Technology Co., LTD Kwai Chung, Hong Kong), Puff Plus (PVG Inc., Wilmington, Delaware, USA), Flum Float (Flumgio Technology LTD, Tuen Mun, Hong Kong), Flum Gio (Flumgio Technology LTD, Tuen Mun, Hong Kong), ELFBAR BC 5000 (VAPEONLY, Shenzhen, China), and PuffXtra Limited (Shenzhen Zhengli Electronics Co., Ltd, Shenzhen, China). The products represented six different flavor categories: ‘Cool Mint’, ‘Mixed Berries’, ‘Aloe Grape’, ‘Blue Razz’, ‘Tobacco’, and ‘Other Fruit Flavors’. Each device was stored at room temperature, and all products were analyzed and used within 2 months of purchase. For each product, we recorded the puff number, fluid volume, power, resistance (when given), and rechargeability.

### Product dissection and E-liquid collection

Each product was dissected as described in detail previously^4,11^. The mouthpiece was first removed using pliers. The tank, atomizer, and battery were removed, analyzed, and photographed. The reservoir sponges were placed in 50 mL conical tubes, centrifuged for 3 min at 3,000 RPM, and the e-liquid was diluted in isopropyl alcohol (1:20 or 1:100) in glass tubes and shipped to Portland State University (Portland, Oregon) for gas chromatography/mass spectrometry (GC/MS) analysis of WS-3 and WS-23 concentrations.

### Gas chromatography/mass spectrometry analysis of WS-3 and WS-23 in popular disposable ECs

The extracted e-liquid was analyzed using previously described GC/MS methods^4^. Each sample (50 µL) was dissolved in 0.95 mL of isopropyl alcohol and shipped overnight on dry ice to Portland State University. A 20 µL aliquot of internal standard solution (2000 ng/µL of 1,2,3- trichlorobenzene dissolved in isopropyl alcohol) was added to each diluted sample prior to analysis. Using internal-standard-based calibration procedures described elsewhere^36^ analyses for 178 flavor-related target analytes, two synthetic coolants, and nicotine were performed with an Agilent 5975C GC/ MS system (Santa Clara, CA). A Restek Rxi-624Sil MS column (Bellefonte, PA) was used (30 m long, 0.25 mm id, and 1.4 µm film thickness). A 1.0 µL aliquot of the diluted sample was injected into the GC with a 10:1 split. The injector temperature was 235 °C. The GC temperature program for analyses was 40 °C hold for 2 min, 10 °C/min to 100 °C, then 12 °C/min to 280 °C and hold for 8 min at 280 °C, and then 10 °C/min to 230 °C. The MS was operated in the electron impact ionization mode at 70 eV in the positive-ion mode. The ion source temperature was 220 cc, and the quadrupole temperature was 150 cc. The scan range was 34 to 400 amu. Each of the 181 (178 flavor chemicals, 2 synthetic coolants, and nicotine) target analytes were quantitated using the authentic standard material.

### Gas chromatography/mass spectrometry analysis of WS-23 in exposure systems

The method described above was used to quantify WS-23 deposited into each well in the VitroCell cloud chamber during exposures. A solution of 4.5 mg/ml of WS-23 dissolved in PBS- (phosphate buffered saline minus calcium and magnesium) was loaded into an Aerogen nebulizer (AG-AL1100, San Mateo, California) and aerosolized for a total of two puffs. Aerosol was deposited in the wells of a VitroCell cloud chamber and collected in isopropyl alcohol. Samples were sent to Portland State University for GC/MS analysis.

### EpiAirway culture and exposure

EpiAirway (AIR-100-PE12), a ready-to-use 3-D mucociliary microtissue model of normal human respiratory epithelium, was used for exposures. EpiAirway, which was derived from tracheal/bronchial cells from a healthy non-smoker male, recapitulates the in vivo phenotype, and is cultured at the ALI. EpiAirway microtissues were shipped on cell culture inserts by Mat-Tek Corp (Ashland, MA) in agarose shipping medium. Upon receipt, tissues were activated by replacing shipping medium with EpiAirway Maintenance Medium (AIR-100-MM), then incubated overnight at 37 °C and 5% CO_2_/95% relative humidity, according to the Mat-Tek protocol.

Exposure of EpiAirway to WS-23 aerosols was done in a VitroCell cloud chamber (VITROCELL 12/12 base module, Walkirch, Germany). EpiAirway maintenance medium was equilibrated to 37 °C in each well of the cloud chamber for 15 min before exposure. Aerosols were generated using an Aerogen nebulizer. 200 µL of either PBS- (phosphate buffered saline minus calcium and magnesium) or 4.5 mg/mL of WS-23 dissolved in PBS-, was loaded into the nebulizer to generate an aerosol without heating the solution. EpiAirway were exposed to one puff of either PBS- or WS-23 aerosol, returned to the incubator for 4 h, then exposed to a second puff of PBS- or WS-23 aerosol, after which they recovered in the CO_2_ incubator for 24 h. Exposures were performed with four independent EpiAirway inserts, and three inserts were used for proteomics analysis. An incubator control group was included to compare back to the PBS controls.

After 24 h, tissues were lysed using RIPA buffer (Catalog #89,900, ThermoFisher). The concentration of protein in each lysate was determined using a BCA kit (Pierce BCA Protein Assay Kit, catalog #23,327), and lysates were analyzed at the UCLA Proteomics Core using label-free proteomics analysis. Culture medium was also collected after the 24-h recovery period and used for IL-6 ELISAs.

### Proteomics: sample digestion

Samples were analyzed at the UCLA Proteome Research Center. In brief, 50 µg protein aliquots from each sample were mixed with denaturing buffer (8 M Urea, 0.1 M Tris–HCl pH 8.5), then each aliquot was reduced and alkylated via sequential 20-min incubations with 5 mM TCEP (tris(2-carboxyethyl)phosphine) and 10 mM iodoacetamide at room temperature in the dark while being mixed at 1200 rpm in an Eppendorf thermomixer. 10 µl of carboxylate-modified magnetic beads (CMMB also known as SP3)^37^ were added to each sample. Ethanol was added to a concentration of 50% to induce protein binding to CMMB. CMMB were washed 3 times with 80% ethanol then resuspended with 50 µl of 50 mM TEAB (Triethylammonium bicarbonate buffer). The protein was digested overnight with 0.1 µg LysC (New England Biolabs, P8109S) and 0.8 µg trypsin (Pierce, 90,057) at 37 °C.

Following digestion, 1 ml of 100% acetonitrile was added to each sample to increase the final acetonitrile concentration to over 95% to induce peptide binding to CMMB. CMMB were then washed 3 times with 100% acetonitrile, and the peptide was eluted with 50 µl of 2% DMSO. Eluted peptide samples were dried by vacuum centrifugation and reconstituted in 5% formic acid before analysis by LC–MS/MS.

### Proteomics: LC–MS acquisition and analysis

Peptide samples were separated on a 75 µM ID, 25 cm C18 column packed with 1.9 µM C18 particles (Dr. Maisch GmbH HPLC) using a 140-min gradient of increasing acetonitrile concentration and injected into a Thermo Orbitrap-Fusion Lumos Tribrid mass spectrometer. MS/MS spectra were acquired using Data Dependent Acquisition (DDA) mode.

MS/MS database searching was performed using MaxQuant^38^ (1.6.10.43) against the human reference proteome from EMBL (UP000005640_9606 HUMAN Homo sapiens, 20,874 entries). The search included carbamidomethylation on cysteine as a fixed modification and methionine oxidation and N-terminal acetylation as variable modifications. The digestion mode was set to specific cut by trypsin and allowed a maximum of 2 missed cleavages. The precursor mass tolerances were 20 and 4.5 ppm for the first and second search, respectively, while a 20-ppm mass tolerance was used for fragment ions. Datasets were filtered at 1% FDR at both the PSM and protein-level. Peptide quantitation was performed using MaxQuant’s LFQ mode. The other MaxQuant settings were left as the default.

Statistical analysis of MaxQuant label-free quantitation data was performed with the artMS Bioconductor package^39^ which performs the relative quantification of protein abundance using the MSstats Bioconductor package^40^ (version 3.14.1). If one protein is fully missed in one condition but found in at least 2 biological replicates in the other condition, the MS intensity value will be imputed from the lowest 5 observed MS1-intensity across samples^41^ and both the log2FC and *p* value will be recalculated. Statistically significant changes were selected by applying an imputed log2-fold-change (iLog2FC) (≥ 1.0 or ≤  − 1.0) and an imputed *p* value (iPvalue) (≤ 0.05). Proteomics data have been uploaded to MassIVE Accession number MSV000092524.

### Volcano plots

Volcano plots were made using Prism-GraphPad software (San Diego, CA). Proteins with their corresponding log2(fold change) and − log(*p* value) values were inputted into a table and graphed with log2(fold change) on the x-axis and − log(*p* value) on the y-axis. Differentially expressed proteins were identified as having a − log(*p* value) cutoff greater than 1.3 and were color coded according to downregulation and upregulation based on negative or positive log2(fold change) values.

### Protein ontology

The functional annotation tool from the DAVID Bioinformatics Database^42^ was utilized to analyze proteins that were significantly upregulated or downregulated by ALI exposure of EpiAirway to WS-23 in a cloud chamber. Proteins were selected for analysis based on significance where the cutoff for the ipvalue (adjusted *p* value) $<$ 0.05. The data were sorted numerically into two lists based on ilog2FC, where positive values were upregulated proteins and negative values were downregulated proteins. When proteins could not be identified by DAVID, “view unmapped IDs” was selected. The individual protein ID was then characterized by its protein name, which was provided by the master sheet. The protein name was then inputted into uniprot.org where the updated protein ID could be found. The old ID was then located in the list separated based on the direction of regulation and replaced with the new uniprot ID. This process was repeated until all proteins were identified. The final lists of upregulated and downregulated proteins were then analyzed using DAVID. “UNIPROT_ACCESSION” was selected as the identifier followed by “gene” as the list type, using the gene list manager. The “homo sapiens” filter was applied to limit the annotation to one species. The list was then analyzed using the functional annotation clustering tool. Clusters with an enrichment score ≥ 1.3 were selected as significant with a medium classification stringency filter. The same process was then repeated for the list of downregulated proteins.

### Protein pathways and targets

The full proteomics spreadsheet for the WS-23 versus PBS treatments was uploaded into Ingenuity Pathway Analysis software (IPA) (Qiagen Inc., Germantown, MD. USA), and unidentified proteins were identified using Uniprot ID matching (https://www.uniprot.org/id-mapping). An expression analysis was run using cutoffs set at (− 0.585 to 0.585) Log2 fold change and *p* < 0.05. For all IPA analyses, default settings and cutoffs were used.

### BEAS-2B cell culture and treatment

Human bronchial epithelial cells (BEAS-2B) obtained from the American Type Culture Collection (ATCC) [Manassas, VA] were cultured in BEGM Bronchial Epithelial Cell Growth Medium supplemented with the BulletKit from LONZA (Catalog #CC-3170) [Basel, Switzerland]. Nunc T-25 EasYFLask tissue culture flasks (ThermoFisher Catalog #156,340, Waltham, MA) were coated for 2 h with Basal Medium (BEBM medium), collagen, bovine serum albumin, and fibronectin prior to subculturing. Upon reaching 90% confluency, cells were rinsed with Dulbecco’s phosphate-buffered saline (DPBS) without calcium and magnesium then detached using 1.5 mL of 0.25% trypsin EDTA/DPBS with polyvinylpyrrolidone for 1.5 min at 37 °C. Cells were passage into T-25 flasks at 75,000 cells/flask, and the medium was changed every other day. For all assays, BEAS-2B cells were plated at a density of ~ 21,000 cells/cm^2^ and allowed to attach and expand for 24 h, then treated for different lengths of time depending on the assay.

BEAS-2B cells were treated with WS-23 for various assays. WS-23 was dissolved in BEGM and made fresh for each experiment. For each assay, three independent experiments were performed.

### Phalloidin stain

BEAS-2B cells were plated in Ibidi chamber slides (Ibidi Catalog #80,807, Grafelfing, Germany) and incubated at 37 °C, 5% CO_2_, and 95% relative humidity for 24 h. BEAS-2B cells were treated with WS-23 (4.5, 1.5, 0.45 and 0.15 mg/mL) for 8 h, then rinsed with PBS 3 times, fixed with 4% paraformaldehyde for 10 min, then rinsed again 2 × with PBS. Cells were then labeled with 1 × phalloidin-iFluor 594 (catalog #ab176757) [Abcam, Cambridge, United Kingdom]) for 1 h, following the manufacturer’s recommended procedure. Cells were viewed with a Nikon Eclipse Ti inverted microscope (Nikon Instruments, Melville, NY, USA) using a 60X objective, and images were collected using an Andor Zyla VSC-04941 camera (Andor, Belfast, UK) at exposures that did not produce saturation. Images were deconvoluted on the fly using Nikon Elements software.

### BioStation live cell imaging assay

Non-invasive morphology analysis of live cells was performed using a 20 × phase contrast objective in a BioStation CT with automatic Z-focus^43^. After attachment, BEAS-2B cells were treated with WS-23 (0.45, 1.5, 4.5 mg/mL) dissolved in BEGM cell culture medium. Images were taken every 2 h for 48 h to collect time-lapse data for analysis. Images of BEAS-2B morphology in the untreated control were compared to the treated groups for the 12th, 24th, 36th, and 48th hour time points. The concentrations of WS-23 used in the motility assay have been confirmed to efficiently dissolve in cell culture media^4^.

### Gap closure assay

Ibidi gap closure 2-well inserts (Catalog No 80209) were placed in 12 well plates, one plate was used for the control group and another plate was used for the treatment group. BEAS-2B cells were seeded at a density of 75,000 cells/well of the inserts. Cells grew for 24 h, and the insert was removed to create a gap. BEGM was added to the control plate and WS-23 (0.45 mg/ml, 0.15 mg/ml, and 0.045 mg/ml) was added to the treatment plate.

The plates were loaded into a BioStation CT for 3 days and imaged every 3 h with a 10 × phase contrast objective. The images produced by the BioStation CT were uploaded and stitched into time-lapse videos capturing the full 68-h span using CL Quant. A green mask, created by our lab to specifically identify BEAS-2B cells, was loaded into the CL Quant procedures menu and applied to the video. The mask was edited frame by frame to assure that no debris was misidentified with the mask. A measurement procedure was applied which calculated the total area of the cells in each time frame. These measurements were entered into GraphPad prism.

### ELISAs

IL-6 concentration in cell media samples was determined using the ELISA MAX Deluxe Set Human IL-6 kit (Catalog #430504 [Biolegend, California, USA]). The assay was performed according to their manufacturers’ protocols from BioLegend.

### Statistical analysis

For the IL-6 ELISA, IL6 concentrations were quantified in media from EpiAirway tissues treated with WS-23 or PBS- in the VitroCell or in the incubator control group. Data are the means ± standard deviations of four independent experiments. Means of PBS and WS-23 treated groups were compared to the incubator control group using a one-way ANOVA followed by Dunnett’s multiple comparison test (*p* < 0.05).

For the gap closure data, changes in area with respect to time and concentration were compared to the untreated control and analyzed using a two-way ANOVA followed by Dunnett’s multiple comparison test (*p* < 0.05).

### Supplementary Information

Supplementary Information.

## Data Availability

Proteomics data is available on MassIVE database (https://massive.ucsd.edu/ProteoSAFe/static/massive.jsp). Dataset: MSV000092524.
