# Supplementary material for: A synthetic coolant (WS-23) in disposable electronic cigarettes impairs cytoskeletal function in EpiAirway microtissues exposed at the air liquid interface
Source: Sci Rep. 2023 Oct 7;13:16906. doi: 10.1038/s41598-023-43948-4 (PMC10560211; doi:10.1038/s41598-023-43948-4)

A Synthetic Coolant (WS-23) in Disposable Electronic Cigarettes Impairs Cytoskeletal Function in EpiAirway Microtissues Exposed at the Air Liquid Interface

Man Wong[1], Teresa Martinez[1], Mona Tran[1], Cori Zuvia[1], Alisa Gadkari[1], Esther E. Omaiye[1], Wentai Luo[2], Kevin J. McWhriter[2], Jihui Sha[3], Ahmad Kassem[3], James Wohlschlegel[3], Prue Talbot[1*]

[1]Department of Molecular, Cell and Systems Biology, University of California, Riverside, CA 92521, United States.

[2]Department of Civil and Environmental Engineering, Portland State University, Portland, OR 97207, United States.

[3]Department of Biological Chemistry, David Geffen School of Medicine at University of California, Los Angeles, CA 90095, United States

Correspondence and requests for materials should be addressed to P.T. (email: talbot@ucr.edu)

Supplementary Table 1: Popular Disposable EC Devices

Device	Puff Number	E-fluid (mL)	Power (mAh)	Rechargeable
Binaries Cabin[1]	10000	20	650	Yes
Zovoo Dragbar 8000[1]	8000	16	630	Yes
Candy King Air	6000	13	600	Yes
Flum Pebble[2]	6000	14	600	Yes
ELFBAR BC 5000[1]	5000	13	650	Yes
Juice Head 5K	5000	14	650	Yes
KK Energy	5000	12	850	Yes
SWFT Mod	5000	15	400	Yes
Truly Bar[1]	5000	13	650	Yes
Dragbar	5000	13	500	Yes
Hyppe Max Air 5000	5000	13	650	Yes
Hyde Retro Rave	5000	10	400	Yes
Kros Nano	5000	13	650	Yes
Hyde IQ	5000	8	500	Yes
Air Bar Box 5K[1]	5000	14	650	Yes
Lost Mary	5000	13	650	Yes
Hyde Mag[1]	4500	10	500	Yes
NKD 100 Max	4500	10	500	Yes
Kros Mini	4000	10	650	Yes
Real Floom Infinity	4000	8	1100	No
ELFBAR BC 3500	3500	10.5	650	Yes
Pod Mesh FLO	3500	10	1000	No
Flum Float[1]	3000	8	1100	No
Flum Gio[2]	3000	8	800	No
7 Daze Egge	3000	7	400	Yes
VaporLax	3000	6.5	1000	No
Air Bar Box X NKD 100	3000	10	1500	Yes
Juice Head Bars	3000	8	650	Yes
Puff XTRA Limited	3000	8	1000	No
ESCO Bar	2500	6	1000	No
BLVK Ello	2500	7	850	No
Sirius 2200	2200	10	1500	No
Hyppe Max Flow	2000	6	900	No
Ignite V15	1500	5	850	No
Gang XL	600	2	450	No

EC devices from websites which compiled tier lists of “most popular disposable ECs from 2022”. Device names and corresponding puff numbers, e-fluid capacity (mL), power(mAh), and rechargeability (provided by the manufacturer).

[1] Indicates devices that were listed on multiple websites.

[2] Indicates devices that were not on any of the website lists but were included in the table.

Supplementary Table 2: WS-23 Concentrations in Analyzed Disposable EC Products

Device	WS-23 (mg/mL)
PP Cool Mint	40.1
FF Cool Mint	33.1
PP Lychee Ice	31
PbP Tangerine Ice	29.9
PP Banana Ice	29.3
Puff XL Cool Mint	29.3
PP Mixed Berries	28.8
PP Blueberry Ice	28.7
PP Blue Razz Ice	24
PbP Peach Ice	24
Elf BC5000 Tropical Rainbow Blast	23.6
FF Aloe Mango Melon Ice	18.6
Elf BC5000 Blue Razz Ice	18.3
FF Aloe Grape	18.2
FF Mixed Berries	17
Puff XL Aloe Grape	16.8
FF Blue Raspberry Ice	16.8
PbP Mixed Berries	15.5
Elf BC5000 Mango Peach	14.9
FG Tobacco Cream	9.6
Puff XL Tobacco	6.2
PP Tobacco	1

Supplementary Table 3: WS-3 Concentrations in Analyzed Disposable EC Products

Device	WS-3 (mg/mL)
FF Cool Mint	10.9
FF Aloe Mango Melon Ice	5
FF Mixed Berries	4.2
PbP Tangerine Ice	2
Puff XL Cool Mint	Trace
FG Tobacco Cream	Trace
Elf BC5000 Mango Peach	Trace
Puff XL Aloe Grape	Trace

Supplementary Figure 1: ELFBAR atomizers

ELFBAR Tropical Rainbow Blast with plastic atomizer (right) compared to ELFBAR Blue Razz Ice atomizer (left)

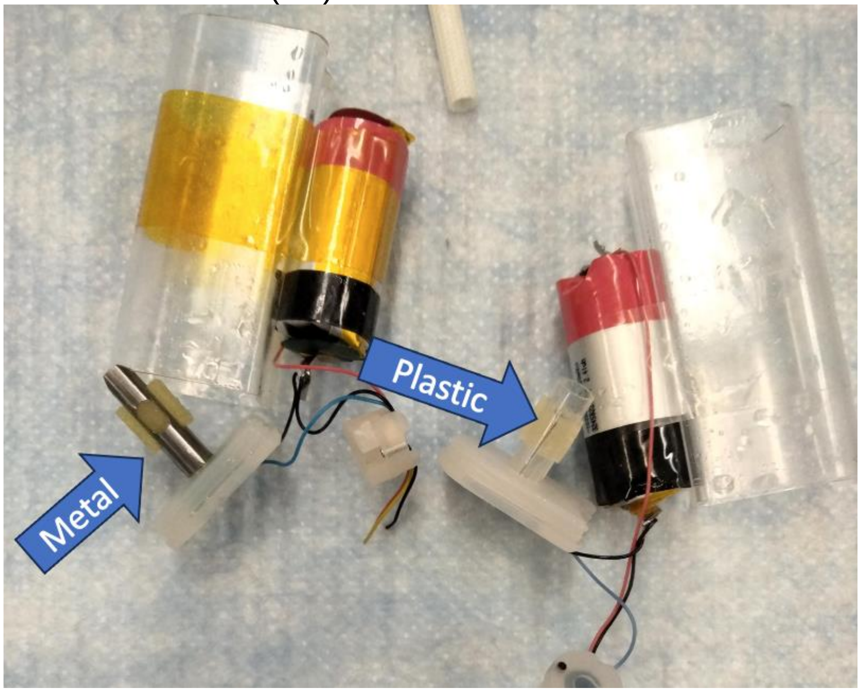

Supplementary Figure 2: Flum Float Images

(Left to Right) Flum Float Cool Mint, Mixed Berries, Aloe Mango Melon Ice, Blue Razz

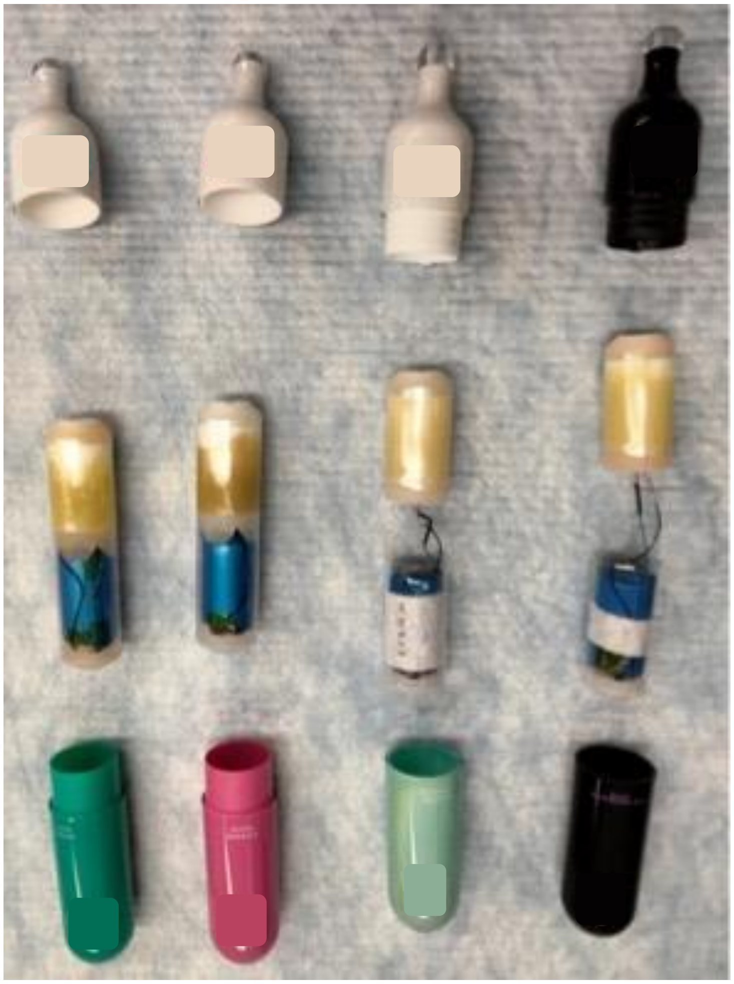

Supplement: Supplementary file 1 — Supplementary Information. [file 41598_2023_43948_MOESM1_ESM.pdf]
